# Analysis of costs in implementing the HEARTS hypertension program in Nigerian primary care

**DOI:** 10.1186/s12962-025-00626-8

**Published:** 2025-05-27

**Authors:** Emmanuel Ndenor Sambo, Muhammad Jami Husain, Soumava Basu, Malau Mangai Toma, Sunday Victor Eze, Kufor Osi, Nanlop Ogbureke, Okeoma Erojikwe, Bolanle Banigbe, Andrew E. Moran, Deliana Kostova

**Affiliations:** 1 UKS Consulting, Abuja, Nigeria; 2 Division of Global Health Protection, Centers for Disease Control and Prevention, Atlanta, GA USA; 3 Non-Communicable Diseases Division, Department of Public Health, Federal Ministry of Health and Social Welfare, Abuja, Nigeria; 4 Resolve to Save Lives, Abuja, Nigeria; 5 Resolve to Save Lives, New York, NY USA; 6 Columbia University, New York, NY USA

**Keywords:** Nigeria, HEARTS program, Hypertension management, Nigeria primary health care, Program costs, Cost analysis

## Abstract

**Background:**

The Nigeria Hypertension Control Initiative (NHCI) program, launched in 2020, integrates hypertension care into primary healthcare using the HEARTS technical package, which includes screening, health counselling, and standardized hypertension treatment protocols. This package has been piloted through NHCI in Kano and Ogun States and in the Federal Capital Territory (FCT) Abuja, as part of the Hypertension Treatment in Nigeria (HTN) project.

**Objective:**

To assess the costs of scaling up the HEARTS hypertension control package and compare these costs with those of usual care.

**Methods:**

Data on the costs of implementing the HEARTS program were collected from 15 purposively sampled primary health facilities in Kano, Ogun, and FCT Abuja between February and April 2024. Costs included training, medicines, provider time, and administrative expenses. We used the HEARTS costing tool, an Excel-based instrument, to collect and analyze the annual costs from a health system perspective, using an activity-based approach.

**Results:**

The estimated annual cost of implementing HEARTS was USD 16 per adult primary care user (PCU), with variations across the three locations: USD 21 in Abuja, USD 11 in Kano, and USD 16 in Ogun. Average annual medication costs per patient treated under HEARTS also varied by location, amounting to USD 28 in Abuja, USD 27 in Ogun, and USD 16 in Kano. Under usual care, annual medication costs per patient were estimated at USD 32 in Kano and USD 16 in Ogun (data for Abuja were unavailable). Major cost drivers for the HEARTS package included provider time (49%) and medication (47%), compared to usual care, where medication alone accounted for 80% of costs. Implementing HEARTS requires a full-time equivalent of 0.45 doctors, 1.59 nurses, and 5.21 community health workers per 10,000 primary care users.

**Conclusions:**

In the Nigerian primary care setting, provider time costs and medication costs emerge as major considerations in scaling up hypertension services. Policy options could consider reducing follow-up visit frequency for well-controlled patients to decrease provider time costs. Additionally, medication costs may be reduced by prioritizing first-line treatments and volume-driven purchasing as program scale-up continues.

## Background

Cardiovascular diseases (CVDs) are the leading cause of death worldwide, and many of these deaths can be prevented by treating hypertension, the silent cause of more than 10 million global deaths annually [1]. Over the past three decades, hypertension has become a major burden in low- and middle-income countries (LMICs) in addition to high-income countries (HICs) [2]. Compared with HICs, a smaller proportion of hypertension patients in LMICs have their blood pressure controlled < 140/90 mmHg [3]. In Nigeria, hypertension is the most frequently diagnosed CVD risk factor, with one in three persons being hypertensive and accounting for almost two-thirds of all CVD deaths in 2019 [4]. Currently, only 11% of Nigerian hypertension patients have their blood pressure controlled [5]. Nigeria has set national targets to control hypertension [4], but the current system does not adequately address hypertension management and control at the primary health care (PHC) level, resulting in hypertensive patients having to seek costly and inconvenient care in secondary or tertiary level semi-urban and urban health facilities [6, 7].

The HEARTS technical package was introduced in 2016 as a strategic framework for CVD prevention at the primary care level. This comprehensive package encompasses evidence-based guidelines for implementing a primary care approach to CVD management, with a specific focus on systematic screening and management of CVD risk factors including lifestyle modifications and standardized pharmacological treatment protocols targeting metabolic risk factors such as hypertension, diabetes, and hyperlipidemia, following a team-based care model [8]. The Nigeria Hypertension Control Initiative (NHCI) introduced in 2020, is spearheaded by the Non-Communicable Diseases Division of the Federal Ministry of Health and Social Welfare (FMoHSW) and supported by Resolve to Save Lives [9]. NHCI serves as a model for the implementation of the HEARTS program in Nigeria. Beyond doctors and nurses, the NHCI approach empowers community health workers to participate in team-based care, thereby increasing the critical mass of health workers providing hypertension care at the PHC level. It has been used to pilot the HEARTS approach to hypertension care in 104 primary health centers in two states, Kano and Ogun, enrolling about 35,000 patients. In addition, 60 clinics in the Abuja Federal Capital Territory (FCT) are also participating as part of a University of Abuja clinical research study of hypertension outcomes.

Future plans to scale up the NHCI program depend on understanding the costs involved. In this study, we present a detailed cost assessment of implementing the hypertension component of the HEARTS program across two Nigerian states and the Abuja Federal Capital Territory (FCT). Our analysis draws upon program cost data obtained from five representative healthcare facilities within each state and the FCT. Understanding the cost drivers for hypertension prevention and treatment programs within Nigeria's primary care system provides valuable insights for policymakers. It informs budgetary requirements for service delivery, identifies cost drivers and opportunities for cost optimization, facilitates population-level scaling, evaluates program cost-effectiveness, and shapes informed health insurance strategies.

## Methods

### Study setting

A PHC center in the Nigerian public sector health system serves a catchment population between 10,000 and 30,000 [10, 11]. Until 2019, PHC facilities were only permitted to screen and refer hypertensive cases to higher levels of care, such as secondary and tertiary health facilities. In 2020, Nigeria introduced the Nigeria Hypertension Control Initiative (NHCI) with the aim of integrating hypertension care and treatment in primary healthcare facilities [9].

We collected primary care level costs from 15 health facilities- five each in Kano, Ogun, and the FCT Abuja- between February and April 2024. These health facilities were purposively selected from all implementing sites (i.e., 104 sites in Kano and Ogun and 60 clinics in the Abuja Federal Capital Territory) based on two equally weighted performance indicators: percentage of patients lost to follow-up and percentage of patients achieving blood pressure control. From each state, the top two, bottom two, and one mid-performing facility were chosen for the study based on their rankings. The facilities in Ogun and Kano implemented the NHCI spear-headed by the NCD Division of the Federal Ministry of Health, while those in Abuja were implementing the Hypertension Treatment in Nigeria Study implemented by the University of Abuja. All three states implemented a standardized hypertension care protocol—the simplified HEARTS protocol involving four stepwise treatment regimens. In Abuja, participating facilities did not offer hypertension care prior to the start of the NHCI program, and thus did not provide information on usual care hypertension treatment approaches. In Kano and Ogun, participating facilities provided comparative data on both usual-care and HEARTS approaches. Since Abuja facilities are part of a clinical research study, they incorporated a higher frequency of patient follow-up visits (i.e., monthly visits for all participating patients) than facilities in Kano and Ogun.

### Patient and public involvement

The study did not involve patients or the public in this research’s design, conduct, reporting, or dissemination plans.

### Hypertension treatment: usual care

Before the introduction of the NHCI in 2020, the management of hypertension was mainly carried out by the physicians in tertiary and some secondary healthcare centers, with little or no systematic management at the PHC facility level [12, 13]. The National Standing Orders for Community Health Workers in Nigeria which delineate protocols for diagnosis and patient care, restricted these workers to conducting screenings and referring patients to higher levels of care for hypertension management. This was inconsistently implemented and interpreted differently by different actors in the primary healthcare space [14]. Usual care practices for the management of hypertension in primary health care centers, which include BP screening frequently followed by referral to secondary or tertiary facilities, are not guided by any standardized treatment guideline. This situation allows PHC workers who do not refer identified cases to higher-level facilities, to rely on residual knowledge from their pre-service training and experience.

### HEARTS hypertension management program

The HEARTS technical package was developed to support ministries of health in enhancing risk-based CVD prevention in PHC settings, particularly in resource limited contexts [15]. The practical step-by-step modules of HEARTS focus on healthy lifestyle counseling, evidence-based treatment protocols, access to essential medicines and technology, team-based care, and systems for monitoring [8]. The comprehensive range of HEARTS implementation activities includes training staff in delivering standard screening, counselling, and protocol-based treatment; record keeping and reporting; ensuring an adequate supply of necessary drugs, introducing patient monitoring tools and reporting systems; and establishing a mechanism for a patient referral from primary care to secondary and tertiary care. As of 2024, 38 countries had implemented the HEARTS package for hypertension control, with 22.9 million hypertension patients enrolled and receiving care in over 184,000 HEARTS PHC facilities [16, 17].

**Fig. 1:**
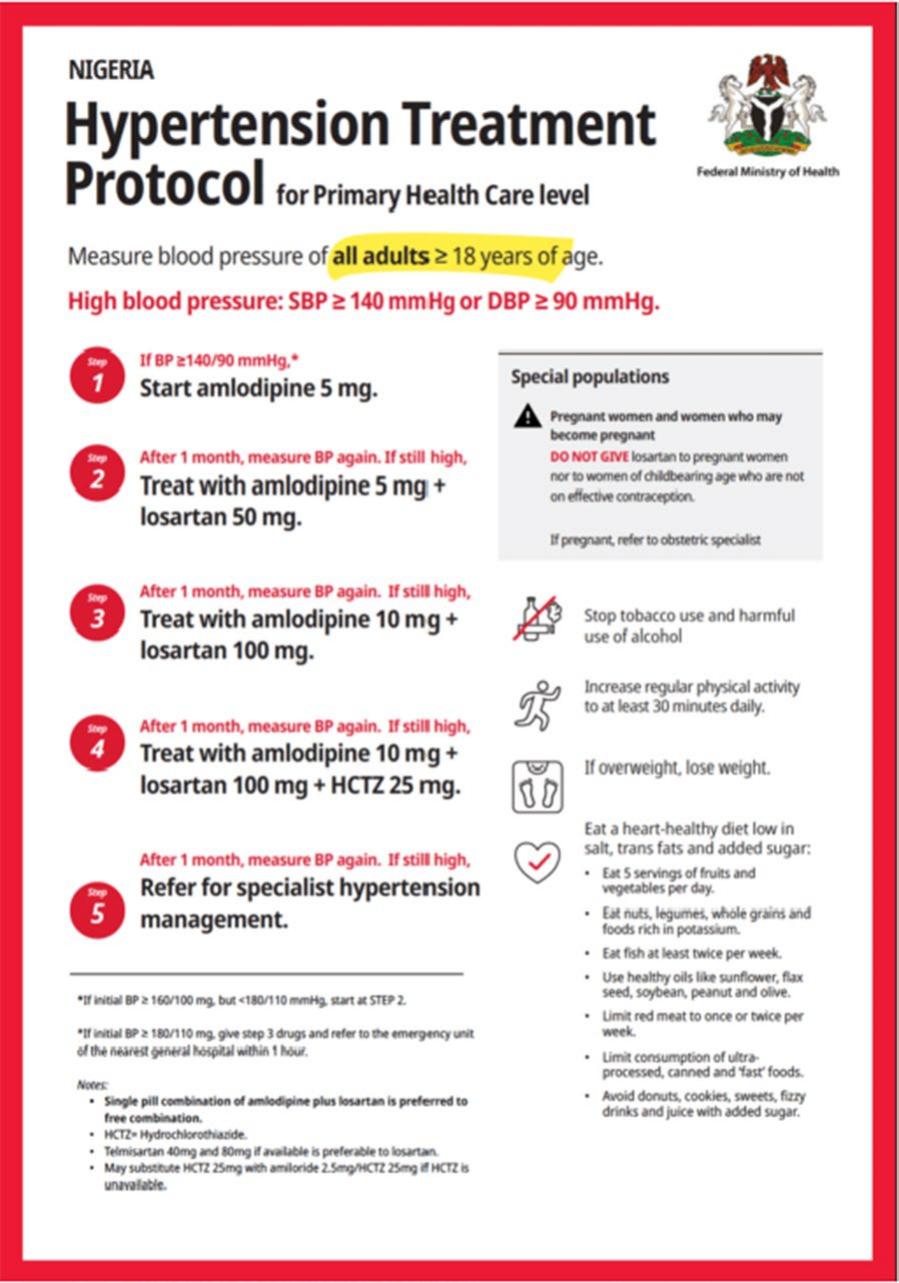
HEARTS standardized hypertension pharmacological treatment protocol in Nigeria. [12] Source: DHIS2: Combating hypertension in Nigeria with decentralized testing, real-time data analytics and a standardized patient-centered treatment protocol

Guided by the HEARTS hypertension protocol, the clinical management protocol for adults with hypertension in Nigeria (defined as systolic blood pressure (SBP)/diastolic blood pressure (DBP) ≥ 140/90 mm Hg) entails a first line of treatment with amlodipine 5 mg daily; a second line of treatment using amlodipine 5 mg plus losartan 50 mg daily; a third line of treatment using amlodipine 10 mg plus losartan 100 mg and a fourth line of treatment using amlodipine 10 mg plus losartan 100 mg plus hydrochlorothiazide 25 mg daily (Fig. 1).

The prescribed medicines are usually generic and are sold to patients at public health facilities. To provide continuous care more sustainably and to reduce the burden on physicians, a team-based care strategy is also being implemented, with nurses and CHWs taking up most of this task. The package also includes practice facilitation support through training and continuous supportive supervision to the participating health facilities.

The HEARTS package in Nigeria is implemented in two states (Kano and Ogun) and the Abuja FCT. It focuses on screening and managing hypertension but does not include other HEARTS components, such as screening and managing diabetes and raised cholesterol. As a result, an integrated CVD risk management scenario has not been carried out and was not analyzed by this study.

### Data sources

In addition to primary data sources including health facility records and registers and other secondary sources, key informants involved in the data collection process included the officers in charge of health facilities, NCD/hypertension focal persons in the health facilities, as well as individuals within the state’s ministries of health and the FCT Primary Healthcare Board. Stakeholders within the NCD Division of the Federal Ministry of Health and Social Welfare were also consulted to provide context.

### HEARTS costing tool

Program costs were assessed using the HEARTS costing tool, a Microsoft Excel-based instrument to collect, track, and evaluate the incremental annual cost of implementing the HEARTS program using activity-based costing from the health systems perspective. The tool allows a detailed assessment of the costs of implementing and operating the HEARTS program at the district or regional level in primary care settings in low- and middle-income countries. The tool is robust and adaptable to local contexts and has been validated through cross-country costing studies, producing reliable and context-specific analyses. The tool is organized by HEARTS package components, so that after entering program costs and other inputs such as population coverage, risk factor prevalence, treatment coverage by protocol steps, provider time, and planned provider numbers, the cost calculations are distributed across different HEARTS modules [18]. See Appendix Table 6 for the major cost components of the HEARTS costing tool. In the Nigerian context, the relevant components include: H (healthy-lifestyle counseling and training cost); E (provider time cost to administer evidence-based treatment protocols); A (hypertension medication and treatment related supplies cost); T (cost-savings from shifting tasks traditionally performed by doctors to nurses and community health workers); and S (administrative and technical personnel cost to keep up the systems for monitoring). The R (risk-based CVD assessment) component is not present in the Nigerian context.

### Data

Appendix Table 7 presents the prevalence of major CVD risk factors and cost inputs used to populate the HEARTS costing tool for each of the two states and the FCT. In Nigeria, the most prevalent risk factor reported is hypertension, with its prevalence ranging between 30.2% in the Northwest geopolitical zone and 32.2% in the North Central geo-political zone [19]. Another highly prevalent risk factor is physical inactivity (27.10%) [20]. Hazardous use of alcohol ranged between 0.2% in Kano and 31.42% in Ogun [21]. Prevalence of the use of tobacco products ranged between 2.15% in Kano and 3.23% in Abuja [22]. Nigeria’s Hypertension Prevention and Management Guidelines [23] classifies hypertension according to BP levels with Grade 1 SBP between 140–159 mmHg or DBP between 90–99, Grade 2 SBP between 160-179 mmHg or DBP between 100–109 mmHg and Grade 3 SBP ≥ 180 mmHg or DBP ≥ 110 mmHg. Among hypertension patients in the health facilities across the states, the proportion of grade 1 hypertension ranged between 25% in Ogun State and 81% in Kano, grade 2 hypertension ranged between 18% in Kano and 63% in Ogun while grade 3 hypertension ranged between 1% in Kano and 12% in Ogun.

The local currency was converted to US dollars using Nigeria’s government’s official exchange rate projection of 435.57NGN/USD for the year 2023 budget [24]. Medicine prices were obtained from health facility pharmacy stores, while personnel costs were obtained from the Consolidated Health Salary Structure (CONHESS) [25] and the Consolidated Medical Salary Structure (CONMESS) [26] for Ogun and Kano, while salary information for health workers in Abuja were obtained from the FCT Primary Health Care Board.

We employed a micro-costing, activity-based methodology in this study. Health workers reported the time spent with patients on each activity in the hypertension management cascade. Then, we multiplied that estimate by each provider's compensation to get the annual provider cost. With overtime and yearly leave excluded, we estimated that every employee puts in 2082 h a year on average. Using the HEARTS protocol in Kano and Ogun, PHC attendees with low-, medium-, and high- CVD risk typically have follow-up visits 2, 5, and 11 times annually, respectively. Usual care practices in the two states reported different figures for follow-up visits averaging 2, 3, and 6 follow-up visits for low, medium, and high risks respectively. But regardless of how serious their ailment was, all patients in Abuja had to make a monthly visit to the medical institution. The prevalence rates of CVD risk categories were proxied using the proportions of different grades of hypertension among patients. Other costs included the cost of training, program management and supportive supervision. We obtained information on treatment protocols, provider time and time-share, and follow-up visit frequency from facility-level key informant interviews and records. In the HEARTS program scenario, based on the time spent with patients on different activities by provider type, on average, the task distribution among provider types was as follows: CHWs handled 70%, nurses 24%, and physicians 6% of the total workload. In the usual care scenario, CHWs managed 81%, nurses 19%, and physicians 2% of the tasks. The total annual program cost, the annual cost per primary care user (PCU), and the annual cost of treatment (medicines) per patient treated for hypertension were assessed in USD and local currency (NGN). The data retrieved from registers and other facility sources corresponds to the year 2023 and the costs represent annual cost.

## Results

### Population coverage

The 2023 total adult population above 18 years in the two states and the FCT is 13,415,364, of which 6,573,528 (49%) are primary care users (Table 1). The total number of people eligible to receive screening, counselling, diagnosis, and treatment under the HEARTS package in the two states and Abuja was obtained by multiplying the total adult population by the primary care attendance rate. The primary care attendance rate is the estimated proportion of adults who are expected to attend a primary care facility in any given year under normal circumstances. We refer to this population receiving health care services in public sector primary health care facilities as the primary care users (PCU). Calculated from Table 1, the total estimated number of people eligible for counselling is 6,573,528, and the total estimated number of people with hypertension that would be receiving treatment with medications was 2,116,676.

**Table 1:** Population coverage

	Abuja	Kano	Ogun
Adult population (18 + years)	2,933,323	7,501,662	2,980,379
Adult primary care users^a^	1,437,328	3,675,814	1,460,386
Providing brief counseling			
Eligible to receive brief advice	1,437,328	3,675,814	1,460,386
Tobacco user	45,995	79,765	36,802
Harmful alcohol	153,794	7,352	458,853
Physical inactivity	389,516	996,146	395,765
Attending patients with hypertension			
Hypertension	462,820	1,183,612	470,244

^a^ PC users (primary care users) are all adults expected to present at the primary care facility regardless of hypertension status

### Hypertension management program cost

Table 2 reports the estimated annual costs, in 2023 USD and NGN, of implementing the HEARTS hypertension management program in Abuja, Kano, and Ogun States. The total annual cost across the three HEARTS programs sums up to USD 93.7 million. This translates to an average of USD 16 per PCU, with variations across the three states—i.e., USD 21 in Abuja, USD 11 in Kano, and USD 16 in Ogun, as shown in Table 3. Annual medication costs per patient treated under the HEARTS program also varied; USD 28, USD 27 and USD 16 in Abuja, Ogun and Kano, respectively, translating to an average of USD 24 per patient treated in the HEARTS program (Table 3). With usual care in the NHCI states (Kano and Ogun) the annual costs per PCU was estimated at USD 12 and USD 7 respectively, while the annual medication costs were estimated at USD 32 and USD 16, respectively (Table 3). Usual care cost estimates for Abuja were not available since no hypertension treatment was provided at the participating facilities prior to the HEARTS initiative.

**Table 2:** Total annual cost of HEARTS and usual-care hypertension control programs in three districts

	Abuja		Kano				Ogun			
	HEARTS		HEARTS		Usual Care		HEARTS		Usual Care	
Number of PC users	1,437,328		3,675,814		3,675,814		1,460,386		1,460,386	
	NGN	USD	NGN	USD	NGN	USD	NGN	USD	NGN	USD
H: Healthy Lifestyles	141,301,795	324,407	630,604,975	1,447,770	207,794,938	477,064	471,232,738	1,081,876	136,565,625	313,533
H1: Training costs	12,696,675	29,150	90,338,135	207,402	0	0	59,273,077	136,082	0	0
H2: Brief counselling costs	128,605,119	295,257	540,266,840	1,240,367	207,794,938	477,064	411,959,661	945,794	136,565,625	313,533
H2.1: Tobacco	10,037,473	23,044	39,442,231	90,553	15,170,089	34,828	17,007,509	39,047	5,638,030	12,944
H2.2: Alcohol	33,562,799	77,055	3,669,045	8,424	1,411,171	3,240	212,053,941	486,842	70,296,395	161,389
H2.3: Physical inactivity	85,004,847	195,158	497,155,564	1,141,391	191,213,679	438,996	182,898,211	419,905	60,631,200	139,200
E: Evidence-based Treatment Protocols	7,124,813,237	16,357,447	8,444,447,619	19,387,119	3,259,805,147	7,483,998	4,535,335,719	10,412,415	1,205,352,731	2,767,300
E1: Ask about patient history—provider time	448,101,461	1,028,770	1,552,288,165	3,563,809	564,468,424	1,295,930	787,384,673	1,807,711	223,731,365	513,652
E2: Assess via physical exam and diagnostic tests—provider time	268,860,877	617,262	1,552,288,165	3,563,809	1,128,936,848	2,591,861	224,967,050	516,489	111,865,683	256,826
E3: Return visits—Counsel and treat per protocol—provider time	6,407,850,899	14,711,415	5,339,871,289	12,259,502	1,566,399,876	3,596,207	3,522,983,996	8,088,215	869,755,683	1,996,822
A: Access to Essential Medicines and Technologies	5,644,652,999	12,959,233	8,409,163,502	19,306,113	16,192,190,290	37,172,156	5,131,439,824	11,780,976	2,987,606,791	6,858,603
A1: Hypertension medications	5,642,234,999	12,953,681	8,392,951,502	19,268,892	16,176,218,290	37,135,487	5,123,939,824	11,763,757	2,980,346,791	6,841,935
A2: Diagnostic tech., machines & supplies	2,418,000	5,551	16,212,000	37,220	15,972,000	36,669	7,500,000	17,219	7,260,000	16,668
T: Team-based care (Savings from training nurses and CHWs to do Doctors' work)*	− 5,589,431,697	− 12,832,453	− 686,013,969	− 1,574,980	− 264,763,238	− 607,855	− 361,029,459	− 828,867	− 105,879,015	− 243,082
T1: Savings from training nurses	− 1,412,713,506	− 3,243,367	− 84,867,806	− 194,843	− 32,754,253	− 75,199	− 147,517,529	− 338,677	− 26,469,780	− 60,770
T2: Savings from training CHWs	− 4,176,718,191	− 9,589,086	− 601,146,163	− 1,380,137	− 232,008,985	− 532,656	− 213,511,930	− 490,190	− 79,409,235	− 182,311
S: Systems for monitoring	27,497,770	63,131	158,565,892	364,042	0	0	75,806,065	174,039	0	0
S1: Human resources	27,497,770	63,131	158,565,892	364,042	0	0	75,806,065	174,039	0	0
Total Program Cost (H + E + A + S)	12,938,265,800	29,704,217	17,642,781,988	40,505,044	19,659,790,376	45,133,219	10,213,814,346	23,449,306	4,329,525,147	9,939,436

*Team-based care leads to cost savings, represented as negative values

**Table 3:** HEARTS Hypertension Program in Nigeria: total cost per PC user and medicine cost per treated patient

	Abuja HEARTS		Kano HEARTS		Kano Usual		Ogun HEARTS		Ogun Usual		HEARTS (Average)
	NGN	USD	NGN	USD	NGN	USD	NGN	USD	NGN	USD	USD
Total Cost per PC user*	9002	21	4800	11	5348	12	6994	16	2965	7	16
Medication cost per person treated with medications for hypertension^**^	12,191	28	7158	16	13,795	32	11,618	27	6758	16	24

*Denominator is total primary care users given in Table 1. ** Denominator is total attending patients with hypertension given in Table 1

Task sharing for hypertension management (i.e., activities conducted by non-physician staff compared to physicians only) would save USD 15.2 million, obtained by adding component T for all three HEARTS programs (Table 2). Out of this, USD 3.8 million come from empowering nurses to take on counselling, screening and treating patients and USD 11.5 million from the task delegation to the community health workers (Table 2).

**Fig. 2:**
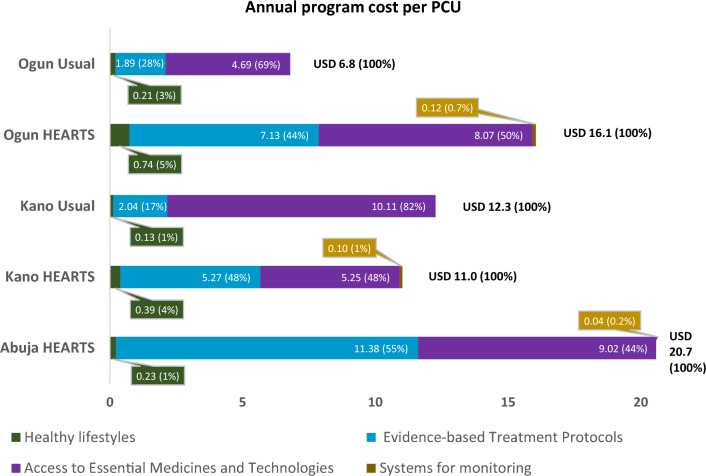
Annual cost in USD (%) per primary care user

Figure 2 breaks down annual costs in USD (%) per PC user while comparing the disaggregated costs by state and between the HEARTS and Usual Care approaches.

The annual costs per PCU in the HEARTS scenario were USD 11, USD 16, and USD 20.7 in Kano, Ogun, and Abuja, respectively. In Abuja, the HEARTS program incorporates a much higher number of visits (i.e., 12 visits per annum) irrespective of patients’ hypertension severity, which contributed to higher provider costs and, hence, the total per PCU cost. The annual per PCU cost for component E (provider time costs) in Abuja was USD 11.38, compared to USD 7.13 and USD 5.27 in Ogun and Kano HEARTS programs. The annual per PCU medicine cost (i.e., A component) in the HEARTS programs were USD 8, USD 5.3, and USD 9 in Ogun, Kano, and Abuja, respectively. The annual per PCU costs in the usual care scenario were USD 6.8 and USD 12.3 in Ogun and Kano, respectively.

Table 4 provides a cross-state comparison of medicine prescription patterns and medicine costs. The average annual HEARTS medication cost per patient in Kano is much cheaper as a large share of patients in the state (81%) had their hypertension managed using only the first line of treatment, amlodipine 5mg as compared to 25% of patients in Ogun (see Appendix Table 7). As a result, a cheaper medication like amlodipine 5mg is prescribed in larger proportion in Kano (99%) than in Ogun (88%). On the contrary, more expensive medicines like losartan (combined with amlodipine) are prescribed in higher proportion in Ogun (75%) compared to Kano (19%). In Ogun, the larger share of patients at higher protocol steps with more expensive medicines prescribed results in higher average medication cost per patient under HEARTS than usual care.

**Table 4:** Medicine prescription patterns

State (program)	Medicine	Unit price (USD)	Dose per day	Percent of patients who received medicine
Abuja (HEARTS)	Amlodipine 5 mg	0.03	1	44
	Amlodipine 5 mg + Losartan 50 mg	0.09	1	45
	Amlodipine 10 mg + Losartan 100 mg	0.18	1	11
	Medication cost per person treated for hypertension (USD/year)	28		
Kano (HEARTS)	Amlodipine 5 mg	0.025	1	99
	Amlodipine 10 mg	0.03	1	1
	Losartan 50 mg	0.10	1	18
	Losartan 100 mg	0.15	1	1
	Medication cost per person treated for hypertension (USD/year)	16		
Kano (Usual)	Amlodipine 5 mg	0.025	1	21
	Lisnopril 5 mg	0.11	1	21
	Methyldopa 250 mg	0.02	3	28
	Bendroflothiazide 2.5 mg	0.10	1	9
	Nifedipine 20 mg	0.16	1	20
	Medication cost per person treated for hypertension (USD/year)	32		
Ogun (HEARTS)	Amlodipine 5 mg	0.028	1	88
	Amlodipine 10 mg	0.03	1	12
	Losartan 50 mg	0.06	1	63
	Losartan 100 mg	0.08	1	12
	Medication cost per person treated for hypertension (USD/year)	27		
Ogun (Usual)	Methyldopa 250 mg	0.020	3	31
	Amiloride-hydrochlorothiazide 10 mg	0.08	1	16
	Nifedipine 20 mg	0.16	1	7
	Medication cost per person treated for hypertension (USD/year)	16		

The annual medication cost per person treated for hypertension (USD/year) is the weighted average of percent of patients who received different medicines (see also, Table 3)

With usual care, prescription patterns varied across health facilities and states and were not guided by a standardized treatment protocol. In Kano state, 28% of patients with hypertension were administered methyldopa pills 3 times a day, resulting in 85% of the total medication share. The methyldopa share was even higher, at 91%, in Ogun state (3 times a day for 31% of patients). Other medicines prescribed under usual care in Kano include lisinopril (21%), nifedipine (20%), and bendroflothiazide (9%). In Ogun, other medicines include amiloride-hydrochlorothiazide (16%) and nifedipine (7%).

Table 5 highlights the time needed from health providers to provide care at the health facility level at full scale-up in the FCT and two states. To fully scale up the implementation of the HEARTS hypertension programs to meet the population in need in the FCT and two states, the full-time equivalent of 298 doctors (168 in Abuja, 57 in Kano, and 73 in Ogun) would be required. Additionally, 1045 nurses (429 in Abuja, 225 in Kano, and 391 in Ogun), and 3428 CHWs (1268 in Abuja, 1594 in Kano, and 566 in Ogun) would be required. This entails 0.45 FTE doctors, 1.59 FTE Nurses, and 5.21 FTE CHWs would be required per 10,000 primary care users.

**Table 5:** Hypertension control: estimated health provider personnel time

	Abuja HEARTS		Kano HEARTS		Kano Usual		Ogun HEARTS		Ogun Usual	
Number of PC users	1,437,328		3,675,814		3,675,814		1,460,386		1,460,386	
	Minutes of provider care	Converted to FTE	Minutes of provider care	Converted to FTE	Minutes of provider care	Converted to FTE	Minutes of provider care	Converted to FTE	Minutes of provider care	Converted to FTE
Health Personnel										
Doctor	20,995,589	168	7,077,168	57	2,709,718	22	9,048,538	73	0	0
Nurse	53,511,875	429	28,084,031	225	10,838,874	87	48,815,764	391	8,803,059	70
CHW	158,209,022	1,268	198,928,550	1,594	76,775,357	615	70,654,395	566	26,409,117	211

FTE (full-time equivalent) is the estimated number of full-time equivalent staffs needed to deliver hypertension care

## Discussion

This study estimates the cost of implementing the HEARTS program in two states and the FCT, which have heterogenous profiles in terms of CVD risk factors, and health system composition, especially around the critical human resources required at the PHC level. This study, therefore, provides insights on the cost of scaling up the HEARTS program to the full catchment population in the states. It also shows the potential budget impact of utilizing the team-based care module of the HEARTS program, where lower cadre health workers are empowered to take on higher-level roles in the screening and management of hypertension in primary healthcare facilities.

The HEARTS program cost per adult attending the health facilities was estimated to be on average approximately $16, with variations across health facilities and states. This accounts for about 1% of the country’s 2023 GDP per capita [27]. While most of the unit costs are similar across states, the major variations were around medicines prices. The average annual cost of medications per patient treated depends on the prescription patterns across patients. In Kano state, HEARTS facilities managed hypertension in > 80% of hypertension patients using only the inexpensive first-line hypertension medication, amlodipine 5 mg, and prescribed relatively less of a more costly medication, like lisinopril or losartan compared with usual care facilities, resulting in lowering medication costs per patient from an average of USD 32 under usual care to USD 16 under HEARTS. A shift to prescribing more of the Nigeria treatment protocol medicines in Ogun HEARTS facilities, by contrast, led to higher costs relative to usual care facilities mostly because the Ogun usual care facilities more often prescribed the inexpensive drug methyldopa. It should be noted, however, that while methyldopa is recommended for treating the hypertensive disorders of pregnancy, it is not among the antihypertensive drug classes recommended for hypertension treatment by the WHO 2021 hypertension treatment guidelines. The most likely explanation for the high methyldopa prescribing rate in Ogun is that this medicine was the only one available in sufficient quantity in the state, because it was procured for managing hypertension in pregnant women.

The number of recommended follow-up visits also had an impact on the total cost of hypertension management in HEARTS facilities, as the top cost drivers of the HEARTS hypertension control program were the cost of provider time (49%) and medicines (47%). This reflects the emphasis of the HEARTS program on spending time in counselling and screening, in contrast with the usual care practices where medicines accounted for 80% and personnel costs only accounted for 18% of the cost of treating one patient. Reducing the frequency of follow-up visits for well controlled patients can be one way of lowering overall program costs.

The cost analysis, in this study, reveals similarities in cost trends with other HEARTS program cost studies conducted in Mexico, Bangladesh, and Ethiopia [28–30]. Specifically, medication, technologies, and provider time for evidence-based treatment protocols consistently emerge as major cost drivers. However, there are notable differences in the average program cost per person under care (PCU) between Nigeria, Ethiopia, and Bangladesh. Nigeria’s average cost per PCU is higher than Ethiopia’s by more than $10 and Bangladesh’s by $7. The key factor contributing to these cost disparities appears to be higher medication prices in Nigeria compared with these other countries. While Nigeria incurs an average of $24 per patient treated for hypertension, Ethiopia and Bangladesh manage to keep medication costs as low as $9 and $18, respectively. Medication costs could potentially be reduced through volume-driven purchasing of effective and reasonably priced drugs if the program achieves sufficient scale. Additionally, successfully controlling hypertension with first-line treatment medications, as seen in the HEARTS program costs in Kano, could also help manage medication costs.

Although the study did not assess the current human resources for health (HRH) strength in the primary care systems of the FCT and the two states, the analysis revealed population-level scale up of the hypertension management program would require 0.45 FTE doctors, 1.59 FTE nurses, and 5.21 FTE CHWs per 10,000 primary care users. Nigeria’s current HRH profile, an overall density of 1.83 skilled health professionals per 1000 population, may not be able to meet this requirement. Moreover, in resource-limited settings, physicians are not always available at the primary health care (PHC) level. In Nigeria, Primary Health Care Centers are mainly staffed by lower-tier health workers, such as Community Health Extension Workers (CHEWS) [14, 31]. Although utilizing CHWs has been successful for maternal and child health services and some infectious diseases [32, 33], there is a missed opportunity in the Nigerian health system in not fully utilizing CHWs for CVD prevention [34, 35]. In the HEARTS program it was observed that the practice of task sharing was slightly reduced compared to usual care. Our findings point to an opportunity to further close the HRH gap for HEARTS hypertension care more affordably, using the task shifting and sharing approach that equips lower cadre health workers to take on higher roles. With the shortage of medical personnel, exacerbated by the ongoing exodus of doctors and nurses, patients have grown accustomed to receiving care from CHEWS and other lower-tier providers, including for hypertension, building a level of trust in these caregivers. Team-based care has improved access by removing barriers that previously limited CHEWS from managing hypertension cases. Patients now benefit from easier access to treatment. Economically, the availability of hypertension care at PHCs has reduced the need for patients to travel to distant General Hospitals or higher-level centers, saving them time, stress, and additional costs. This shift has significantly eased the burden on patients, increasing acceptance of care at PHCs.

While this study did not explore the clinical effectiveness of HEARTS hypertension control programs in Nigeria in terms of hypertension control or downstream prevented cardiovascular events, it has implications regarding potential approaches to optimize future program costs. For example, encouraging all prescribers to adhere to the HEARTS treatment protocol, thereby achieving economy of scale and lower prices for those medicines, and providing guidance to facility pharmacy managers and government procurement agencies on sourcing and pricing of protocol medicines ensures that exit prices for those medicines are as low as possible. The introduction of multi-month dispensing for patients who have attained BP control with high adherence levels reduces the number of visits and contact with health workers, which drives down the personnel cost.

Limiting the Nigeria HEARTS implementation to hypertension alone, may constitute a missed opportunity for efficiency gains from the deployment of an integrated CVD package that includes the screening and management of diabetes and hyperlipidemia. Integration of additional diseases, such as diabetes and hyperlipidemia, could potentially reduce costs in areas such as duplicative visits, diagnostics, patient history collection, provider time, training, and program management. The ongoing efforts of the Federal Ministry of Health focus on an integration-centered approach that offers dual benefits for both the healthcare system and patients. This strategy involves not only integrating non-communicable diseases such as hypertension and diabetes but also embedding hypertension care within well-established programs, like those for HIV. With the high burden of CVDs in Nigeria, the assessment of intervention cost through the HEARTS technical package is important for policy and decision making. More importantly, determining the most cost-effective intervention design is important in the face of the high burden of CVDs and resource constraints that countries’ health systems face.

This report has some limitations. Due to the paucity of up-to-date data at a granular level, we relied on deductions from older national and regional level surveys to extrapolate data such as risk factor prevalence, and primary care attendance rates. To determine unit prices per personnel, we assumed an even distribution of each cadre on the salary scale. The fluctuations in Nigeria’s exchange rate may also limit the comparability of our estimates. At the time of writing up this report, the Naira’s exchange rate against the dollar was around NGN1500 to 1 USD [36], an over 300% loss in value of the Naira from the Government’s position in the 2023 budget estimate [24]. This alters our estimates, especially when viewed against the backdrop of a 28.9% inflation rate in January 2024 [37] that the costing tool does not account for. Additionally, although this report estimates cost for one year (2023), scale-up costs could vary significantly by year due to several factors, such as the patient care cascade, program delivery capacity, implementation pathways, and price negotiations. These multifaceted considerations would indeed be essential for projecting cost dynamics over multiple years. The strengths of the study lie in its use of the HEARTS costing tool and in its ability to disaggregate costs by function and components, allowing planners and decision makers to iterate various implementation strategies until an optimal strategy is determined.

## Conclusion

HEARTS implementation in Nigeria appears not to be substantially more costly than usual care. Monitoring clinical outcomes of the Nigeria HEARTS programs—improvement in hypertension control and downstream averted cardiovascular disease outcomes–will be necessary for establishing the cost-effectiveness of HEARTS compared with usual care. In the meantime, lessons learned from this costing exercise point toward the next steps to lower the cost of HEARTS implementation in Nigeria, including visit spacing and multi-month prescriptions for stably controlled hypertension patients, tighter adherence to HEARTS protocol prescribing, and market-shaping measures to lower hypertension medication costs.

## Data Availability

Data is provided within the manuscript or supplementary information files.

## Appendix

See Tables 6, 7.

**Table 6:** Major cost components of HEARTS hypertension program in Nigeria

(H) Healthy-lifestyle counseling	H1. Training costs H2. Brief counseling costs H3. Other program costs
(E) Evidence-based treatment protocols	E1. Provider time- Ask about patient history E2: Provider time- Assess vis physical exam and diagnostic tests E3: Provider time- Return visits—Counsel and treat per protocol
(A) Access to essential medicines and technology	A1. Diagnostic tests A2. Hypertension medications A3. Diagnostic technologies and supplies
(T) Team-based care	T1. Savings from task sharing with nurses T2. Savings from task sharing with health extension workers
(S) Systems for monitoring	S1. Human resources S2. Technology S3. Supplies S4. Training

**Table 7:** Input costs, cost parameters, and cost assumptions

Input Description	Units	Abuja HEARTS	Kano HEARTS	Kano Usual	Ogun HEARTS	Ogun Usual
Adult population (18+)	Persons	2,933,323	7,501,662	7,501,662	2,980,379	2,980,379
Primary healthcare attendance rate (annual)	Percent	49.0%	49.0%	49.0%	49.0%	49.0%
Adult population with risk factors						
Use of tobacco products	Percent	3.23%	2.15%	2.15%	2.52%	2.52%
Hazardous or harmful use of alcohol	Percent	10.71%	0.20%	0.20%	31.42%	31.42%
Physical inactivity	Percent	27.10%	27.10%	27.10%	27.10%	27.10%
Hypertension (≥ 140/90 mmHg)	Percent	32.20%	31.90%	31.90%	30.20%	30.20%
Low CVD risk (0 to < 10%)	Percent	44%	81%	81%	25%	25%
Medium CVD risk (10 to < 20%)	Percent	45%	18%	18%	63%	63%
High CVD risk (≥ 20%)	Percent	11%	1%	1%	12%	12%
Annual wage (in BIRR, including benefits)						
Doctors	NGN/year	6,900,000	5,165,246	5,165,246	5,165,246	5,165,246
Nurses	NGN/year	3,600,000	4,787,506	4,787,506	4,787,506	4,787,506
CHWs	NGN/year	3,600,000	4,787,506	4,787,506	4,787,506	4,787,506
Lab technician	NGN/year	3,600,000	4,787,507	4,787,507	4,787,507	4,787,507
Distribution of time spent in						
Counseling patients to change behavior (initial visit)						
Doctors	Percent	9	3	3	7	0
Nurses	Percent	23	12	12	38	25
CHWs	Percent	68	86	86	55	75
Assessing patients (initial visit)						
Doctors	Percent	9	3	3	7	0
Nurses	Percent	23	12	12	38	25
CHWs	Percent	68	86	86	55	75
Follow-up visits						
Doctors	Percent	9	3	3	7	0
Nurses	Percent	23	12	12	38	25
CHWs	Percent	68	86	86	55	75
Screening and Diagnosis						
Approximately how much time (in minutes) does a health provider spend to:						
Screen patients and ask about patients' health history	Minutes/patient	7	13	5	12	4
Counsel patients with behavioral risk factors to change behavior (e.g. quit tobacco, cease using alcohol harmfully, increase physical activity)	Minutes/patient	10	11	4	14	4
Provide a physical exam (including relevant metabolic screenings) to assess patients' total CVD risk	Minutes/patient	6	11	8	4	2
Treatment for High CVD Risk						
How many follow-up visits should a person with the following levels of CVD risk undertake annually?	Visits/patient					
Low CVD risk (≥ 0% to < 10%)		11	2	2	2	2
Medium CVD risk (≥ 10% to < 20%)		11	5	3	5	3
High CVD risk (≥ 20%)		11	11	6	11	6
Approximately how much time (minutes) will the following health providers spend with a patient during a visit?	Minutes/patient					
Doctors/Nurses/HEWs		13	11	5	12	5
LCU to USD exchange rate	NGN/USD	435.57	435.57	435.57	435.57	435.57
Number of health providers in need of training in counseling patients						
Counsel patients to change behavior	Persons	198	1452	-	627	-
Training to counsel patients to change behavior (5A’s)*				-		-
Classroom size	Persons	45	52	-	52	-
Hours of training needed	Hours	12	24	-	20	-
Number of Trainers						
Professional trainer(s)	Persons	2	5	-	4	-
Administrative staff	Persons	2	2	-	2	-
Input costs for training						
Hourly wage						
Professional trainer	NGN/hour	10,000	3250	-	4000	-
Administrative staff	NGN/hour	1667	-	-	3000	-
Additional costs						
Facility rental for training (one day)	NGN /day	400,000	150,000	-	200,000	-
Refreshments	NGN /day	294,000	191,750	-	2000	-
Meals	NGN /day	686,000	324,500	-	3000	-
Per diem for staff	NGN /day	-	-	-	30,000	-
Per diem and/or salary of trainees	NGN /day	-	-	-	20,000	-
Transportation stipend for staff	NGN /day	10,000	10,000	-	-	-
Transportation stipend for trainees	NGN /day	10,000	10,000	-	-	-
Pharmacological treatment for hypertension- Protocol steps^a^						
% of patients in Protocol Step #1	Percent	44%	81%	21%	25%	32%
Medicine name and unit		Amlodipine (5 mg)	Amlodipine(5 mg)	Amlodipine (5 mg)	Amlodipine (5 mg)	Aldomet (125 mg)
Medicine price	NGN/pill	15	11	11	12	8
Times per day × Days per year		1X365	1X365	1X365	1X365	3X365
% of patients in Protocol Step #2	Percent	45%	18%	21%	63%	16%
Medicine name and unit		Amlodipine (5 mg) + Losartan (50 mg)	Amlodipine (5 mg)	Lisnopril (5 mg)	Amlodipine (5 mg)	Moduretic (10 mg)
Medicine price	NGN/pill	40	11	50	12	37
Times per day × Days per year		1X365	1X365	1X365	1X365	1X365
Medicine name and unit			Losartan (50 mg)		Losartan (50 mg)	
Medicine price	NGN/pill		44		25	
Times per day × Days per year			1X365		1X365	
% of patients in Protocol Step #3	Percent	11%	1%	28%	12%	7%
Medicine name and unit		Amlodipine (10 mg) + Losartan (100 mg)	Amlodipine (10 mg)	Aldomet (250 mg)	Amlodipine (10 mg)	Nifedipine(20 mg)
Medicine price	NGN/pill	80	13	8.33	13	70
Times per day × Days per year		1X365	1X365	3X365	1X365	1X365
Medicine name and unit			Losartan (100 mg)		Losartan (100 mg)	
Medicine price	NGN/pill		67		33	
Times per day × Days per year			1X365		1X365	
% of patients in Protocol Step #4	Percent			9%		
Medicine name and unit				Bendroflothiazide (2.5 mg)		
Medicine price	NGN/pill			42.86		
Times per day × Days per year				1X365		
% of patients in Protocol Step #4	Percent			20%		
Medicine name and unit				Nifedipine (20 mg)		
Medicine price	NGN/pill			70		
Times per day × Days per year				1X365		

^a^ HEARTS programs include protocol steps, whereas usual programs have prescription pattern of prescribed medicines

## Abbreviations

BP: Blood pressure
CDC: Centers for Disease Control
CHW: Community health workers
CVD: Cardiovascular Disease
DBP: Diastolic blood pressure
FCT: Federal Capital Territory
FMOHSW: Federal Ministry of Health and Social Welfare
FTE: Full Time Equivalent
HRH: Human resources for health
HTN: Hypertension Treatment in Nigeria
NGN: Nigerian Naira
NHCI: Nigeria Hypertension Control Initiative
PCU: Primary Care User
PHC: Primary Health Care
SBP: Systolic blood pressure
USD: United States Dollars
